# Symposium on visualization in high-performance computing at SIGGRAPH Asia 2015

**DOI:** 10.1007/s12650-016-0381-7

**Published:** 2017-04-27

**Authors:** Fumiaki Araki, Koji Koyamada

**Affiliations:** 1 Japan Agency for Marine-Earth Science and Technology (JAMSTEC), Yokohama, 236-0001 Japan; 2 Academic Center for Computing and Media Studies, Kyoto University, Kyoto, 606-8501 Japan



The symposium on visualization in high-performance computing (VHPC) was held in conjunction with ACM SIGGRAPH Asia 2015 in Kobe, Japan, between 2 and 4 November, 2015. The number of participants was over 260 from Japan and a few other countries. The state-of-the-arts in many aspects of scientific visualization, information visualization and visual analytics were presented and discussed. In total, 45 papers were submitted from five countries. After a rigorous peer review process where each paper was reviewed by at least two reviewers, 15 full papers were selected for presentation. Three speakers were invited by VHPC. As a keynote presentation, Prof. Kwan-Liu Ma from the University of California, Davis (UC Davis), USA, gave a talk titled “Visualization and high performance computing”. Dr. E. Wes Bethel from Lawrence Berkeley National Laboratory (LBNL), USA, presented his talk titled “HPC visualization and analysis at the exascale: big headaches, big opportunities” as an invited talk. Dr. Hirofumi Seo from SCIEMENT Inc., Japan, gave a presentation of his visualization works in medical front as a special talk. In addition, VHPC featured two tutorials and a visualization contest. In the final session, VHPC also featured a panel discussion, including six panelists on the unsolved visualization research problems.

In the symposium, 15 papers were selected for presentation from 45 submissions. Among those presentations, four papers were finally accepted for publication by the standard peer review process of *Journal of Visualization* (JOV). We wish to thank the editors and reviewers of JOV for making it possible to publish this special feature from VHPC. We also thank the authors for their careful and insightful work and cooperation in the preparation of the revised papers. It will be our pleasure if readers appreciate the hot topics in visualization research as a result of this special feature. We would like to express our sincere thanks to the staff at Springer-Verlag for their kind support.

